# Biomimetic piezoelectric hydrogel system for energy metabolism reprogramming in spinal cord injury repair

**DOI:** 10.7150/thno.108329

**Published:** 2025-03-31

**Authors:** Guoliang Shi, Tianqi Su, Junyang Li, Aoao Wang, Gan Gao, Benzhang Tao, Nantian Chen, Lu Tian, Jun Yan, Lingzhou Zhao, Jianning Zhang, Yantao Zhao

**Affiliations:** 1 Senior Department of Orthopedics, the Fourth Medical Center of PLA General Hospital; Beijing Engineering Research Center of Orthopedics Implants, Beijing, 100048, PR China.; 2 Senior Department of Neurosurgery, the First Medical Center of PLA General Hospital; Medical School of Chinese PLA, Beijing, 100853, PR China.; 3 Xijing 986 Hospital Department, The Fourth Military Medical University, Xi'an, 710032, PR China.; 4 Tianjin Medical University, Tianjin, 300052, PR China.; 5 National Center for Orthopaedics; Beijing Research Institute of Traumatology and Orthopaedics; Beijing Jishuitan Hospital, Capital Medical University, Beijing, 100035, PR China.; 6 Air Force Medical Center, The Fourth Military Medical University, Beijing, 100089, PR China.

**Keywords:** piezoelectric hydrogel, spinal cord injury repair, neural stem cells, ATP, neural regeneration

## Abstract

**Rationale:** Spinal cord injury (SCI) leads to limited regenerative capacity and severe energy deficiency in the injury microenvironment. This study aimed to develop a biomimetic piezoelectric hydrogel system that could recapitulate the native tissue microenvironment while enabling wireless physical regulation for SCI repair.

**Methods:** A piezoelectric hydrogel was fabricated by integrating K_0.5_Na_0.5_NbO_3_ (KNN) nanoparticles with porous decellularized spinal cord matrix gel (pDG). The hydrogel's effects on vascular endothelial cell migration, neural stem cell differentiation, and ATP synthesis were evaluated *in vitro*. RNA sequencing was performed to identify key regulatory pathways. The therapeutic efficacy was assessed in a rat model of spinal cord hemisection, examining motor function and angiogenesis.

**Results:** The piezoelectric hydrogel demonstrated excellent biocompatibility and significantly enhanced vascular endothelial cell and neural cell migration. Under ultrasonic stimulation, the hydrogel promoted neural stem cell differentiation into neurons more effectively than control hydrogels. The piezoelectric stimulation increased ATP synthesis and calcium ion flux, activating the Ca2+/Camk2b/PGC-1α signaling axis. *In vivo* studies showed that implantation of the piezoelectric hydrogel combined with ultrasound stimulation significantly improved motor function recovery and promoted angiogenesis.

**Conclusion:** The piezoelectric hydrogel system presents an effective strategy for SCI repair through energy metabolism reprogramming and demonstrates promising potential in neural tissue engineering applications.

## Introduction

Spinal cord injury (SCI) is a devastating neurological condition that severely compromises motor and sensory functions, significantly diminishing the quality of life of affected individuals 1-3. Following an SCI, the disruption of neuronal axons and subsequent neuronal death result in dysfunction of neural circuits 4. The primary goal of injured spinal cord repair is to restore structural connectivity, particularly between neurons and axons. However, the pathophysiological microenvironment that emerges postinjury often impedes the regenerative process, posing a significant challenge 5. Consequently, the creation of a conducive regenerative microenvironment and the utilization of pivotal cell fate regulators are essential strategies for fostering neural regeneration, analogous to the interplay between "soil" and "fertilizer" in nurturing growth.

A significant hurdle in the repair of SCI is suboptimal integration between implanted biomaterials and native spinal cord tissue, which frequently leads to insufficient axonal growth within the graft and ultimately fails to promote nerve regeneration 6, 7. To emulate natural spinal cord tissue more effectively, we employed a decellularized spinal cord matrix (DSCM) hydrogel, designed to closely mirror its biological characteristics. This DSCM hydrogel not only provides the necessary structural scaffolding for tissue regeneration but also acts as a cellular vehicle 8, 9, effectively creating favorable "soil" for the microenvironment at the site of injury. To counteract the slow gelation rate and potential for loss within the body, we integrated porous gelatin methacrylate (pGM), which is known for its rapid light-curing properties and ease of injection 10, 11.

In the context of injured spinal cord repair, an imbalance in energy metabolism is acknowledged as a pivotal factor that impedes nerve regeneration. Neural cells, with their substantial energy requirements, are particularly vulnerable to disruption of the energy supply following injury, which can exacerbate cell death and lead to further functional impairment 12, 13. Consequently, the prompt provision of energy, akin to "fertilizer" for the nervous system, is essential for fostering nerve regeneration. Electrical cues play a distinctive role in nerve regeneration by directly modulating cellular electrophysiological activities and facilitating tissue repair through the regulation of metabolism, gene expression, and protein synthesis 12-15. In recent years, electrical stimulation has demonstrated considerable potential in enhancing nerve regeneration and restoring functional capabilities 16, 17. However, traditional electrical stimulation methods necessitate the use of multiple devices, wired connections, and implantable electrodes 18. In this context, piezoelectric stimulation induced by ultrasound (US) has emerged as a promising clinical technique, offering the benefits of wireless operation and an autonomous energy supply 19, 20. In inorganic piezoelectric crystals, the non-centrosymmetric arrangement of ions within the crystalline lattice enables linearly proportional polarization displacement in response to externally applied mechanical stress. This structural asymmetry induces charge separation at the material boundaries, generating electric potential gradients that directly couple mechanical deformation with electrical field generation, a phenomenon defined as the piezoelectric effect 21. K_0.5_Na_0.5_NbO_3_ (KNN), a biodegradable piezoelectric material renowned for its exceptional biocompatibility and piezoelectric properties, is particularly well-suited for *in vivo* graft applications 22, 23.

In this study, we developed a biodegradable wireless piezoelectric hydrogel tailored for injured spinal cord repair. This innovative hydrogel creates a conducive microenvironment that, through piezoelectric stimulation, prompts nerve cells to produce increased energy, thereby facilitating injured spinal cord repair. This strategy effectively supplies the requisite "soil" and "fertilizer" for nerve regeneration, presenting novel perspectives and therapeutic approaches for the treatment of SCI.

## Results and Discussion

### Preparation and characterization of the hydrogels

**Figure 1:**
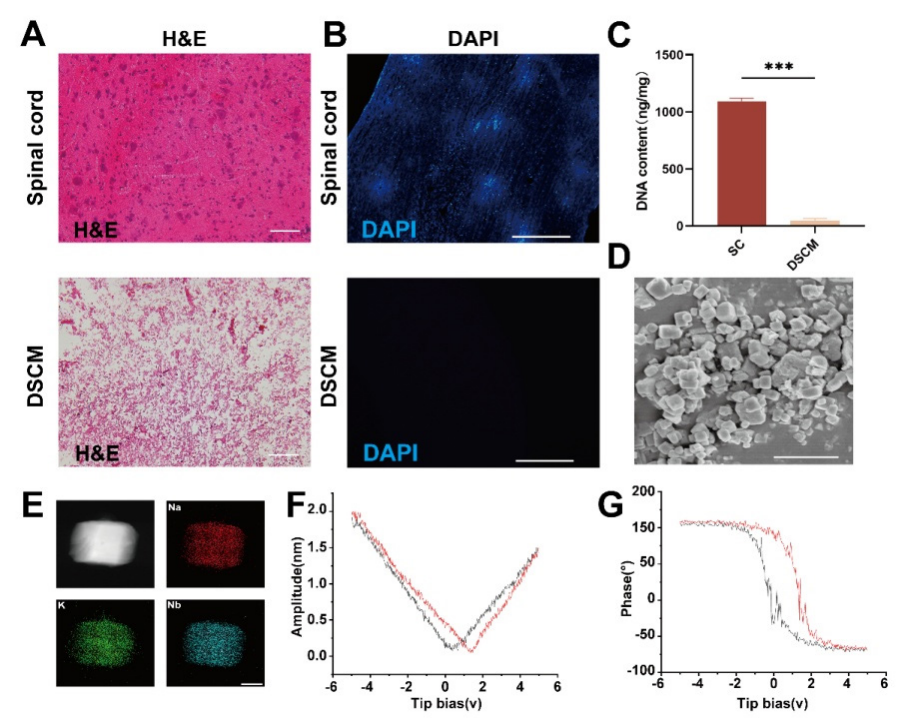
** Preparation and characterization of the DSCM and KNN nanoparticles.** (A) Comparative H&E-stained sections of native and decellularized rat spinal cord tissues, highlighting the reduced nuclear content in DSCM (scale bar = 100 μm). (B) Nuclear staining of native spinal cord tissue and DSCM cells, showing the effectiveness of the decellularization process (scale bar = 1 mm). (C) Assessment of DNA content revealed a significant difference between native spinal cord tissue and DSCM. *p < 0.05, **p < 0.01, ***p < 0.001; n = 3. (D) Micrograph of KNN nanoparticles, demonstrating their morphology and size distribution (scale bar = 1 μm). (E) Elemental analysis of KNN nanoparticles, confirming the presence of key elements and the purity of the sample (scale bar = 1 nm). (F) Amplitude ring curve of KNN nanoparticles, illustrating their piezoelectric response to an applied electric field. (G) Phase ring curve of KNN nanoparticles, revealing substantial polarization reversal indicative of their piezoelectric properties.

Initially, adult rat spinal cords were harvested to fabricate a DSCM. Hematoxylin-eosin (H&E) staining revealed a markedly diminished nuclear presence in the DSCM than in the normal spinal cord (SC) tissue, as depicted in Figure 1A. DAPI staining further confirmed the absence of nuclear signals following decellularization, as shown in Figure 1B. Analysis of the residual DNA content confirmed a significantly lower DNA concentration in the DSCM than in the normal spinal cord tissue, as illustrated in Figure 1C.

The KNN nanoparticles, synthesized via solid-phase sintering, had an average diameter of 804.53 ± 23.41 nm and a zeta potential of 42.4 ± 0.36 mV, as detailed in Figure S1-2. These characteristics indicate excellent dispersion quality. Energy-dispersive X-ray (EDX) analysis revealed potassium (K), sodium (Na), and niobium (Nb) as the predominant elements within the KNN nanoparticles, with no significant presence of impurities, indicating to the high purity of the sample, as shown in Figure 1D-E. Piezoelectric force microscopy (PFM) amplitude curves displayed a distinct butterfly shaped pattern, with amplitude variations that were symmetrically responsive to the applied electric field, as demonstrated in Figure 1F. This indicates a significant and uniform piezoelectric response. The phase curves showed an approximately 180-degree phase inversion under the influence of an electric field, indicating substantial polarization reversal, as shown in Figure 1G. This phenomenon was not observed in the pDG hydrogels, as shown in Figure S3-4. The sharp phase transition corresponds to the rapid reorientation of piezoelectric domains, which is consistent with the material's high-voltage electrical characteristics of the material.

**Figure 2:**
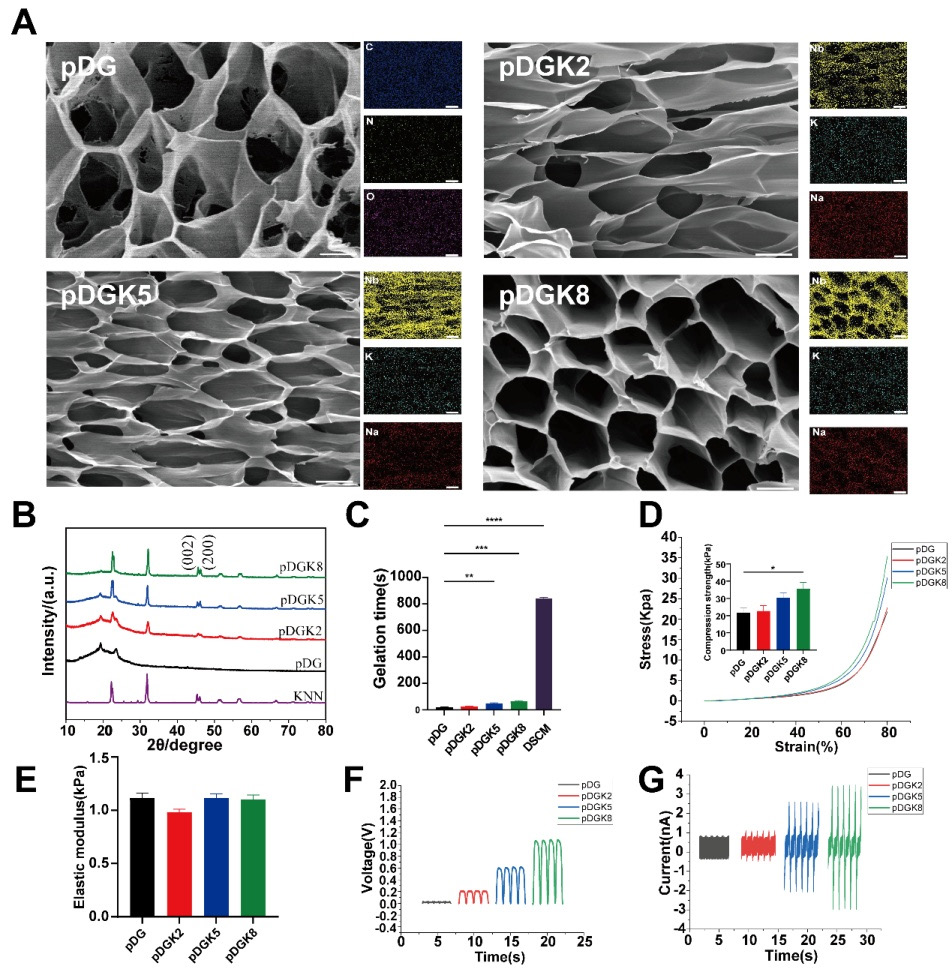
**Preparation and characterization of the piezoelectric hydrogel.** (A) SEM and EDX elemental analysis of the piezoelectric porous hydrogel, showing its microstructure and elemental composition (SEM scale bar = 15 μm, EDX scale bar = 50 μm). (B) XRD pattern of the piezoelectric hydrogel, indicating its crystalline structure. (C) Gelation times for the piezoelectric hydrogel: components pDG and pDGK under UV illumination, and DSCM at 37°C, demonstrating rapid gelation kinetics. **p < 0.01, ***p < 0.001, ****p < 0.0001; n = 3. (D) Stress-strain curve of the piezoelectric hydrogel, with the maximum compressive modulus highlighting its mechanical properties. *p < 0.05; n = 3. (E) Elastic modulus of the piezoelectric hydrogel, illustrating its compliance compared with that of biological tissues. (F) Open-circuit voltage of the piezoelectric hydrogel, reflecting its ability to generate electrical potential in response to mechanical stress. (G) Output current of the piezoelectric hydrogel, showing the electrical output as a function of its piezoelectric properties.

The DSCM underwent lyophilization and pepsin digestion to yield a pregel solution. This solution was then blended with pGM and KNN nanoparticles to form pDG (a composite of DSCM and pGM) and pDGK (a blend of KNN-loaded pGM and DSCM), which were subsequently gelatinized. The KNN concentrations of 0.2% w/v, 0.5% w/v, and 0.8% w/v were designated pDGK2, pDGK5, and pDGK8, respectively. Scanning electron microscopy (SEM) analysis revealed distinct porous structures within the hydrogel (Figure 2A). The incorporation of KNN did not significantly alter the DSCM morphology, likely due to the excellent dispersibility of KNN. Elemental analysis confirmed the uniform distribution of KNN nanoparticles within the hydrogel (Figure 2A). The porous structure of hydrogels reportedly supports neural stem cell migration and differentiation *in vitro*, providing a favorable physical environment 24. Hydrogel degradation is crucial as effective degradation prevents material accumulation, which could impede new tissue growth 25. Therefore, we assessed the degradation properties of the piezoelectric hydrogel (Figure S5). With increasing concentrations of KNN nanoparticles, the degradation rate of piezoelectric hydrogels gradually accelerated, which may be attributed to the nanoparticles affecting the crosslinking between hydrogel networks. By week 4, pDG had a degradation rate of approximately 70.1%, while pDGK8 had a degradation rate of approximately 97.3%. X-ray diffraction (XRD) analysis of the porous composite gel scaffolds and pure KNN nanoparticles revealed two peaks at (022) and (200) at 2θ = 45° (Figure 2B), characteristic of a chalcogenide structure 26, 27. A comparative analysis of gelation times indicated that both pDG and pDGK gelled significantly more rapidly under UV light than did DSCM alone (Figure 2C). This accelerated gelation prevents hydrogel washout from the injury site by cerebrospinal fluid or loss through gaps post implantation, ensuring effective therapeutic application.

We also assessed the elastic modulus and compressive strength of the hydrogels. The results revealed that the elastic modulus of the pDG and pDGK hydrogels was approximately 1 kPa (Figure 2D-E), similar to the range of 0.1 to 3 kPa in the spinal cord, with softer hydrogels favoring stem cell differentiation into neurons 28. To evaluate the electrical properties of the pDGK hydrogels, we used an electrostatic meter and a linear motor to measure the hydrogel voltage. With increasing KNN content, the piezoelectric hydrogel's open-circuit voltage (Voc) increased to 201.66, 578.03, and 1021.01 mV, whereas no significant voltage signal was detected in the pDG group (Figure 2F). Similarly, the output current increased from 0.98 nA to 3.29 nA (Figure 2G). Previous studies have suggested that a voltage of 100 mV promotes neural stem cell differentiation 29.

### Biocompatibility evaluation of the hydrogels

**Figure 3:**
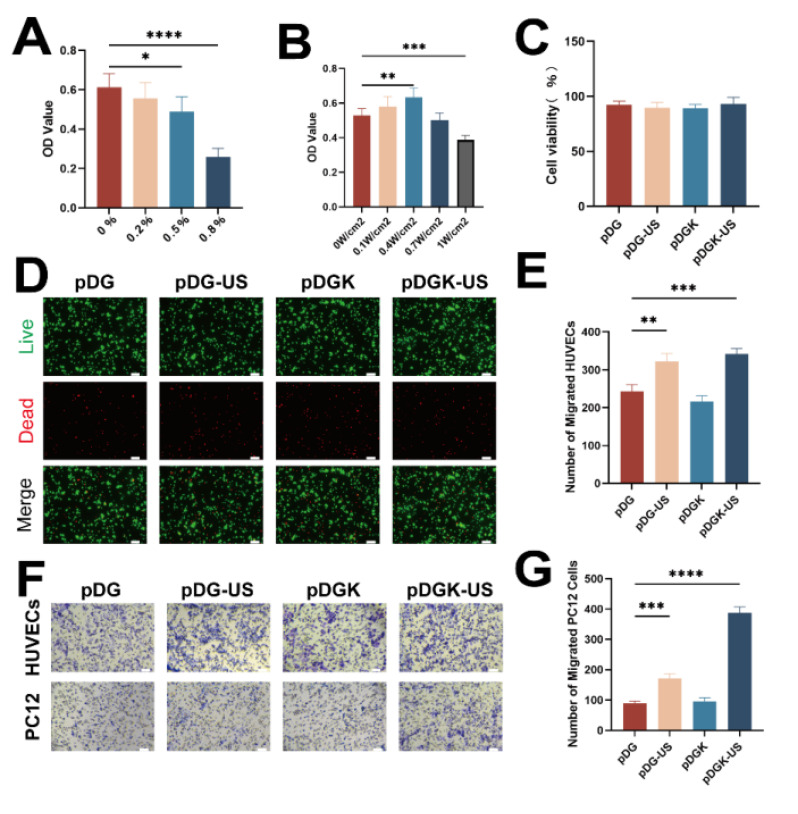
**Evaluation of the biocompatibility and migration efficiency of the piezoelectric hydrogel.** (A) CCK-8 absorbance OD values for cells treated with different concentrations of KNN nanoparticles. *p < 0.05, ****p < 0.0001; n = 3. (B) CCK-8 absorbance OD values for cells treated with 0.2% KNN nanoparticles and different ultrasonic powers. **p < 0.01, ***p < 0.001; n = 3. (C) Assessment of cell viability within the hydrogels under 0.4 W/cm^2^ ultrasonic stimulation and non-stimulated conditions.(D) Live-dead staining images of PC12 cells cultured with the piezoelectric hydrogel, indicating cell viability (scale bar = 50 μm).(E) Quantification of migrated PC12 cells in response to the hydrogels under 0.4 W/cm^2^ ultrasonic stimulation and non-stimulated conditions. *p < 0.05, ***p < 0.001; n = 3. (F) Transwell migration assay results for PC12 cells and HUVECs under piezoelectric stimulation, visualized with crystal violet staining (scale bar = 20 μm). (G) Enumeration of migrated HUVECs in response to the hydrogels under 0.4 W/cm^2^ ultrasonic stimulation and non-stimulated condition. ***p < 0.001, ****p < 0.0001; n = 3.

The biocompatibility of implantable materials is paramount, and we conducted a series of tests to assess this ability. Initially, we cocultured KNN nanoparticles with PC12 cells and observed robust cell viability at a concentration of 0.2% w/v (Figure 3A). However, an increase in the nanoparticle concentration led to a decrease in cell proliferation, potentially because the nanoparticles occupied the space necessary for cell expansion. To align with the characteristics of natural spinal cord tissue, considering factors such as gelation time, morphology, elastic modulus, and piezoelectric properties, we selected pDGK2 for further experiments (referred to as the pDGK group). Additionally, optimizing the ultrasonic power is essential; we determined that an ultrasonic power of 0.4 W/cm² provided optimal outcomes (Figure 3B), aligning with FDA guidelines for ultrasound application 30. Supplementary live‒dead staining further confirmed the excellent biocompatibility of the piezoelectric hydrogel (Figure 3C-D). In all groups, no significant damage or inflammatory lesions were found in major organs, including the heart, lungs, liver, spleen, and kidneys, after 8 weeks of treatment. All these data show that piezoelectric hydrogels have good biocompatibility *in vivo* (Figure S6).

### Enhancing effect of piezoelectric hydrogels on the migration of vascular endothelial and nerve cells

Blood vessels and nerves share an intimate anatomical relationship, with their parallel arrangement forming the neurovascular unit (NVU), which is pivotal to nerve regeneration 31, 32. Recognizing the importance of the NVU microenvironment, we explored the impact of the piezoelectric hydrogel on cell migration, a key process in both vascularization and nerve regeneration. Migration assays demonstrated that the piezoelectric hydrogel significantly promoted the migration of both vascular endothelial and nerve cells (Figure 3E-G). This effect, if replicated *in vivo*, could stimulate vascular growth and nerve cell migration at the site of injury.

### Effect of piezoelectric hydrogels on neural stem cell differentiation

Bioelectricity, as a fundamental biophysical property of living organisms, manifests through long-term stable resting membrane potentials (*V*_m_), ranging from approximately -10 to -90 mV across mammalian cell types 33. These *V*_m_ fluctuations and associated bioelectrical signals play an instructive role in stem cell differentiation and lineage commitment, with hyperpolarization serving as a regulatory trigger to accelerate differentiation 34, 35. The endogenous electric fields (EFs) generated by *V*_m_ gradients further orchestrate tissue repair and wound healing in extensively damaged or regenerating tissues/organs 36, 37.These EFs not only direct stem cell homing by establishing spatiotemporal guidance cues but also orchestrate cellular migration toward injury sites, where functional tissue reconstruction is achieved via coordinated secretory, proliferative, and differentiation programs 38. Furthermore, endogenous EFs mediate intercellular communication among electrically excitable cells, modulating essential physiological processes such as synaptic plasticity and neural network synchrony 39. To harness these electrodynamic principles, we investigated the regulatory effects of piezoelectric signals on neural stem cells (NSCs), aiming to decode their potential in bridging bioelectrical signaling with neuroregeneration.

**Figure 4:**
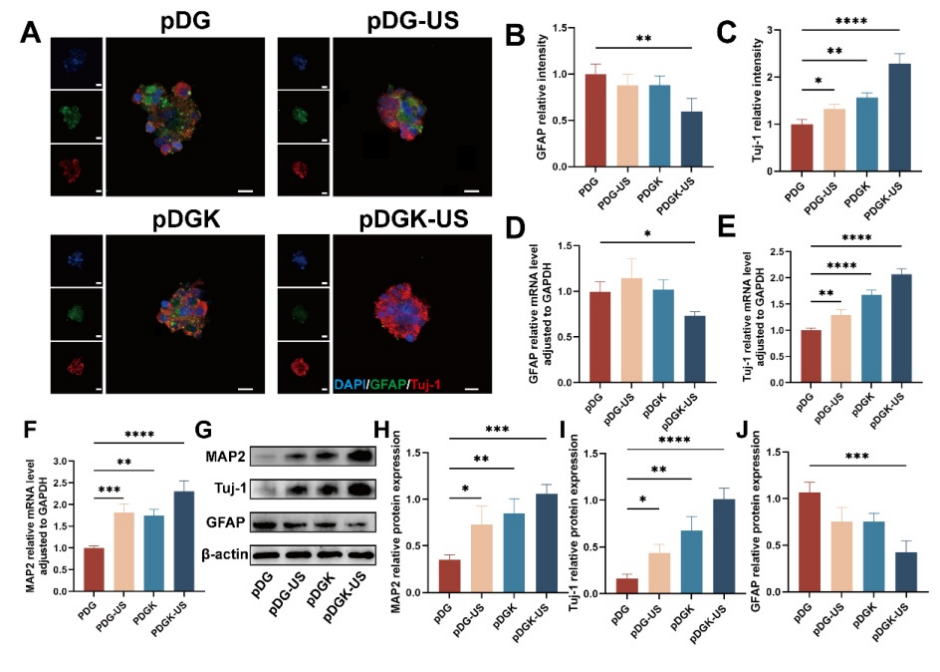
** Effect of piezoelectric hydrogels on neural differentiation.** (A) Neural differentiation under piezoelectric stimulation, with Tuj-1 and GFAP labeled neurons and astrocytes, respectively (scale bars = 50 μm). (B) Analysis of the fluorescence intensity of GFAP-positive cells across different groups. **p < 0.01; n = 3. (C) Analysis of fluorescence intensity in Tuj-1-positive cells across different groups. *p < 0.05, **p < 0.01, ****p < 0.0001; n = 3. (D) Quantitative mRNA expression levels of the GFAP gene in the different groups, were normalized to GAPDH as an internal control. *p < 0.05; n = 5. (E) Quantitative mRNA expression levels of the Tuj-1 gene across groups, were normalized to the level of those of GAPDH, which was used as an internal control. **p < 0.01, ****p < 0.0001; n = 5. (F) Quantitative mRNA expression levels of the MAP2 gene across groups, normalized to those of GAPDH, which was used as an internal control. **p < 0.01, ***p < 0.001, ****p < 0.0001; n = 5. (G) Protein expression of Tuj-1, GFAP, and MAP2 of NSCs cultured on different samples. (H-J) Quantification of Western blot data. *p < 0.05, **p < 0.01, ***p < 0.001; n = 3.

Piezoelectric hydrogels, when subjected to ultrasound, which provides external, wireless mechanical forces, generate electrical signals, thereby modulating neural stem cells (NSCs). For our study, fetal rat NSCs were isolated and utilized for further experimentation (Figure S7). At the same time, the biocompatibility of piezoelectric hydrogel on neural stem cells was further verified (Figure S8) . Given their pluripotency, NSCs can differentiate into neurons, astrocytes, and oligodendrocytes. We used the fluorescent markers - neuronal class III β-tubulin (Tuj-1), microtubule-associated protein 2 (MAP2), and glial fibrillary acidic protein (GFAP), to distinguish between neurons and astrocytes. After 7 days of induction, the piezoelectric group (pDGK-US) presented a significantly greater proportion of NSCs that differentiated into neurons (Figure 4A-C, S9). RT-qPCR analysis confirmed that the gene expression levels of the astrocyte marker GFAP was decreased (Figure 4D), whereas the expression of newborn neurons (Tuj-1) and mature neurons (MAP2) were increased in the piezoelectric group (Figure 4E-F). This is also confirmed by the WB results (Figure 4G-J). In conclusion, the piezoelectric hydrogel more efficiently facilitated NSC differentiation into neurons and concurrently suppressed the formation of astrocytes.

### Mechanisms underlying piezoelectric signaling in promoting NSCs differentiation into neurons

The pro-differentiation effect of piezoelectric signals on neural stem cells (NSCs) is essentially a process of converting mechanical energy (ultrasound) into bioelectrochemical signals through piezoelectric materials, thereby orchestrating intracellular energy metabolism and signaling networks. Specifically, the localized microelectric fields generated by piezoelectric hydrogels under ultrasound stimulation mimic the physiological functions of endogenous bioelectric fields (EFs), regulating energy metabolism and neurogenesis through the following multi-tiered mechanisms.

**Figure 5:**
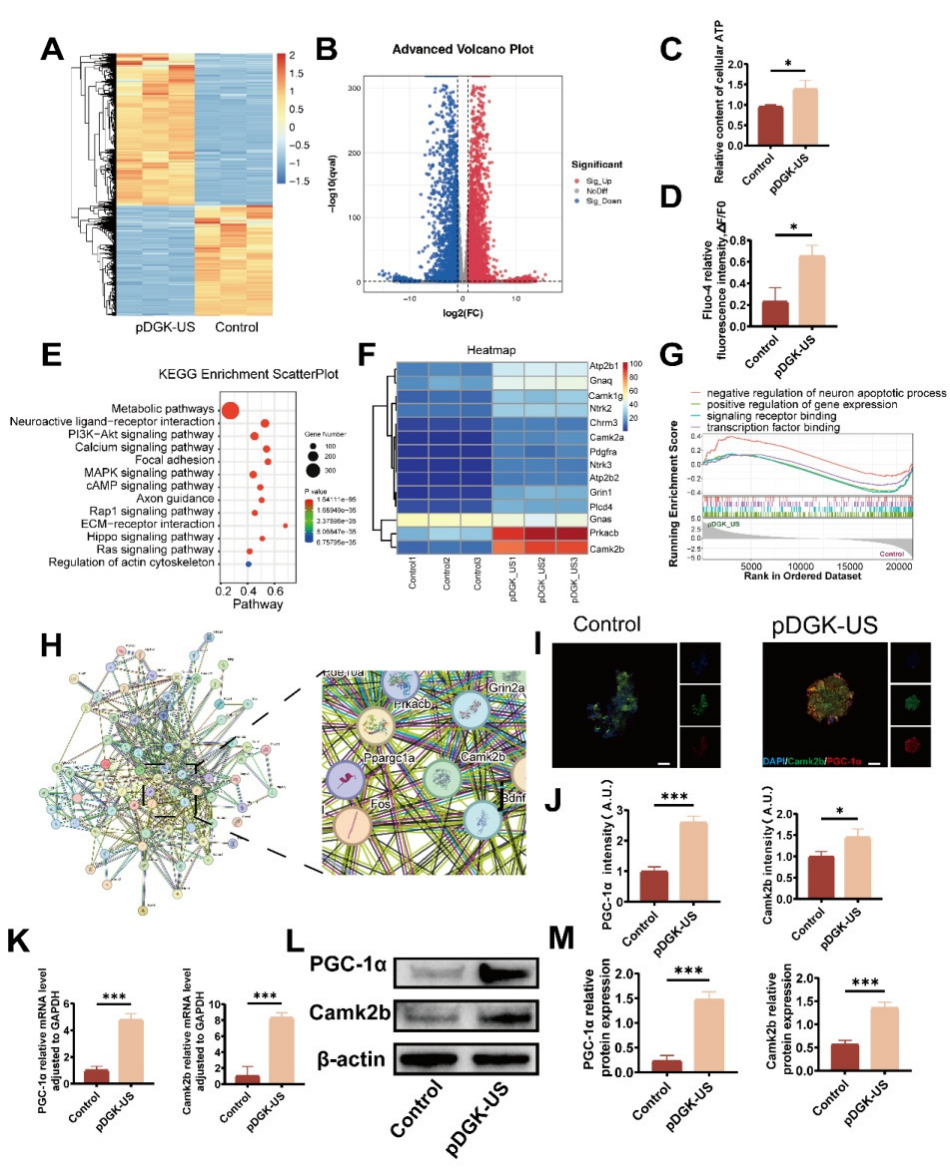
** Bioinformatics analysis and validation of the impact of piezoelectric hydrogels on neural differentiation.** (A) Heatmap depicting differentially expressed genes between the piezoelectric pDGK-US group and the nonpiezoelectric control group, with orange indicating upregulated genes and blue indicating downregulated genes. (B) Volcano plot comparing gene expression in the piezoelectric pDGK-US group to that in the nonpiezoelectric control group, where the x-axis represents log2 (fold change) and the y-axis represents -log10 (P-value). (C) Measurement of the intracellular ATP content relative to that of the control. *p < 0.05; n = 3. (D) Relative fluorescence intensity assessment of cellular calcium influx. *p < 0.05; n = 3. (E) KEGG pathway enrichment analysis highlighting significant pathways. (F) Heatmap showing upregulated genes within the calcium signaling pathway. (G) GSEA indicating the upregulation of PGC-1α-involved pathways. (H) PPI analysis revealing the connections between Camk2b, PGC-1α, and genes associated with neuronal differentiation and axonal extension. (I) Immunofluorescence mapping of PGC-1α and Camk2b positive cells, visualized under microscopy (scale bars = 50 μm). (J) Relative fluorescence intensity of PGC-1α and Camk2b positive cells. *p < 0.05, ***p < 0.001; n = 3. (K) Gene expression of PGC-1α and Camk2b. ***p < 0.001; n = 3. (L) Protein expression of PGC-1α and Camk2b of NSCs cultured on different samples. (M) Quantification of Western blot data. *p < 0.05, **p < 0.01, ***p < 0.001; n = 3.

To elucidate the mechanisms through which piezoelectric signals augment NSC differentiation into neurons, we conducted RNA sequencing (RNA-seq) to capture comprehensive transcriptional profiles. The nonpiezoelectric group (pDG) was utilized as a control, whereas the piezoelectric group (pDGK-US) was subjected to comparative analysis. The correlation coefficients between samples satisfied the experimental criteria (Figure S10). Principal component analysis (PCA) was applied to the gene expression data to uncover disparities in gene expression patterns (Figure S11). Differential gene expression was determined using the criteria |log2fc| ≥ 1 and q < 0.05. Overall, 3,294 genes were upregulated, and 2,386 genes were downregulated, as depicted in the volcano plot and heatmaps (Figure 5A-B). K-means clustering of the top 100 upregulated genes revealed their predominant involvement in neural development (e.g., Tubb3, Dcx, and Ndrg4), cellular synapse and cytoskeleton formation (e.g., Camk2b, Caly, Negr1, and Cd24), and metabolic processes (e.g., Smpd3, Acsbg1, and Nwd1) (Figure S12). Subsequent Gene Ontology (GO) enrichment analysis revealed that the enriched terms across the three categories-biological process, cellular component, and molecular function--were strongly associated with neuronal differentiation, ion channel activity, axon growth, cell migration, and energy metabolism (Figure S13-15). During neurogenesis, NSCs undergo continuous proliferation and differentiation, processes that necessitate significant energy support 40, 41.

We posited that neural differentiation and migration are intricately tied to energy metabolism, with ATP, as the principal energy currency for neuronal cells, being indicative of these metabolic shifts 42. To validate this hypothesis, ATP levels were quantified, revealing that the piezoelectric-stimulated group had a significantly elevated ATP content compared with the control group (Figure 5C). To determine the role of calcium ion influx in triggering these signaling pathways, the Fluo-4 fluorescence intensity was measured. A substantial increase in calcium ions influx was detected following piezoelectric treatment (Figure 5D, S16). The selective uptake of calcium ions by mitochondria enhances ATP synthase activity in axonal mitochondria by optimizing the proton motive force across the inner membrane, a process mediated through the calcium-dependent activation of rate-limiting dehydrogenases (e.g., pyruvate dehydrogenase complex) in the tricarboxylic acid cycle 46.

These findings suggest that piezoelectric stimulation bolsters the energy yield in neurons, potentially driving a cascade of biological responses. Electrical stimulation may enhance transmembrane biosynthesis of adenosine triphosphate (ATP) by activating membrane-bound ATPase activity 43. The hydrolysis energy released from ATP drives conformational transitions and polymerization of monomeric globular actin (G-actin) into filamentous actin (F-actin), thereby enabling energy-dependent regulation of cytoskeletal dynamic remodeling 44. The remodeled cytoskeleton further serves as fundamental structural units participating in diverse cellular life activities.

To identify the signaling pathways involved in this enhancement, Kyoto Encyclopedia of Genes and Genomes (KEGG) analysis was employed. The analysis indicated that the calcium signaling pathway, cAMP signaling pathway, and MAPK signaling pathway were notably enriched (Figure 5E). In conjunction with Gene Ontology (GO) analysis, it was inferred that calcium signals are pivotal in energy metabolism., aligning with the classical mechanism whereby electrical stimulation triggers calcium influx via voltage-gated calcium channels (VGCC) 45.

Further examination of gene expression within the calcium signaling pathway highlighted the significant upregulation of Camk2b, suggesting its pivotal role in these processes (Figure 5F). In the context of nerve regeneration, substantial energy is needed for intricate biological activities such as growth cone regeneration, cytoskeletal rearrangement, and the reestablishment of synaptic and neural circuit connections. These processes rely on healthy axonal mitochondria for energy provision.

Peroxisome proliferator-activated receptor-gamma coactivator (PGC-1α), a critical transcriptional coactivator, governs the expression of nuclear-encoded mitochondrial genes, thus increasing axonal mitochondrial density and bioenergetic capacity 34. Gene set enrichment analysis (GSEA) confirmed the essential function of PGC-1α in neuronal growth and transcriptional regulation (Figure 5G). The protein-protein interaction (PPI) network further corroborated the link between Camk2b and PGC-1α (Figure 5H). Subsequent analysis at both the gene and protein levels confirmed the upregulation of Camk2b and PGC-1α, underscoring their importance as key regulatory factors in the piezoelectric modulation process (Figure 5I-M).

### *In vivo* efficacy of the piezoelectric hydrogels in facilitating injured spinal cord repair

**Figure 6:**
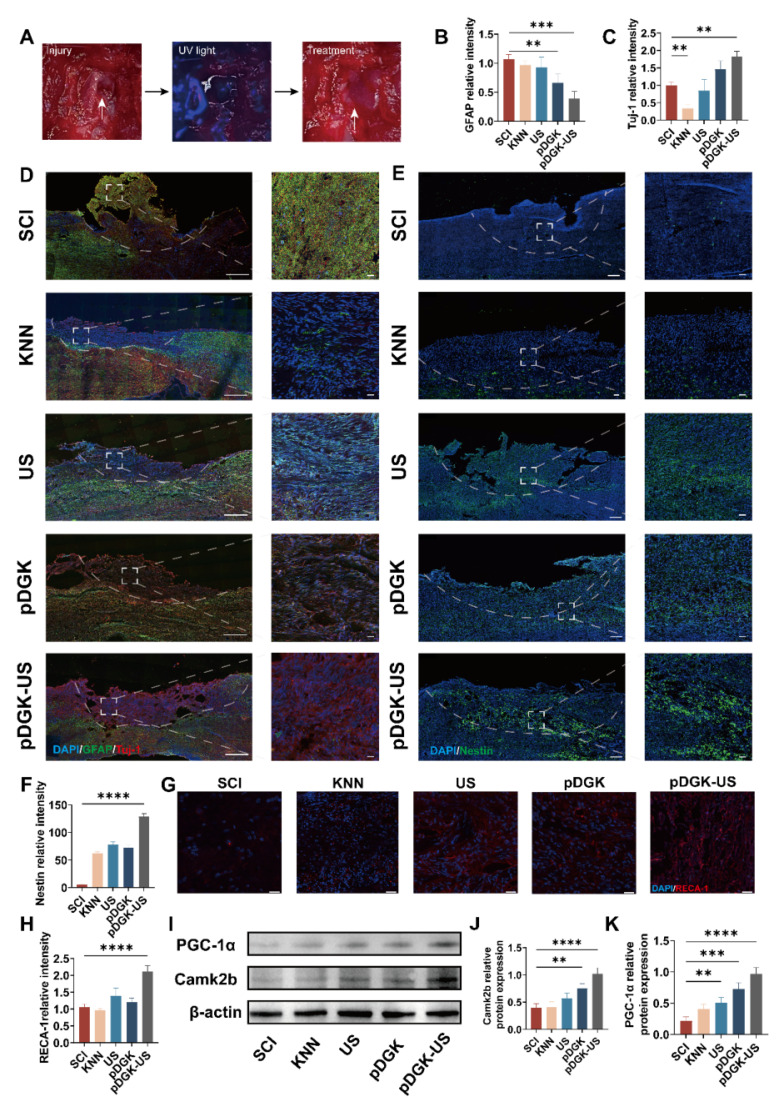
***In vivo* histological examination of piezoelectric hydrogels in injured spinal cord repair.** (A) Schematic representation of the *in vivo* implantation of piezoelectric hydrogels (scale bars = 2 mm). (B) Comparative analysis of the relative fluorescence intensity of GFAP positive cells among the different groups. **p < 0.01, ***p < 0.001; n = 3. (C) Comparative analysis of the relative fluorescence intensity of Tuj-1 positive cells among the different groups. ***p < 0.001; n = 3. (D) Fluorescence images showing Tuj-1 and GFAP positive cells at the injury site for each group, at 8 weeks post-surgery (scale bars = 50 μm). (E) Fluorescence images showing Nestin positive cells at the injury site for each group, at 1 week post-surgery (scale bars = 50 μm). (F) Comparative analysis of the relative fluorescence intensity of Nestin positive cells among the different groups. ****p < 0.0001; n = 3. (G) Fluorescence images of RECA-1 positive cells at the injury site for each group at 8 weeks post-operatively (scale bars = 20 μm). (H) Comparative analysis of the relative fluorescence intensity of PGC-1α positive cells among different the groups. ****p < 0.0001; n = 3. (I) Protein expression of PGC-1α and Camk2b of damaged tissue on different samples. (J) Quantification of Camk2b Western blot data. **p < 0.01, ****p < 0.0001; n = 3. (K) Quantification of PGC-1α Western blot data. **p < 0.01, ***p < 0.001, ****p < 0.0001; n = 3.

We evaluated the *in vivo* efficacy of the piezoelectric hydrogel in facilitating injured spinal cord repair via a rat model of spinal cord hemisection. 8-week-old female Sprague-Dawley (SD) rats with induced spinal cord injury were systematically allocated into five experimental groups (n = 10 per group): 1) SCI control group, 2) KNN nanoparticles treatment group, 3) ultrasound-only group, 4) pDGK hydrogel group, and 5) combined pDGK hydrogel with ultrasound intervention group (pDGK-US).Upon UV light irradiation, the piezoelectric hydrogel rapidly polymerized, effectively preventing the washout by cerebrospinal fluid or tissue fluid, and minimizing the compromise in effectiveness due to gaps (Figure 6A). Given the importance of spinal cord histological recovery for functional restoration, we performed histological immunofluorescence to evaluate the changes in the injured spinal cord after intervention.

The formation of glial scars acts as a persistent barrier to axonal regrowth and significantly impairs functional rehabilitation during the chronic phase of SCI 50. Compelling evidence from both rodent models and human clinical studies has consistently demonstrated the irreversible nature of these astroglial proliferations 51. Consequently, targeted inhibition of glial scar pathogenesis emerges as a crucial determinant for neural circuit reorganization and the restoration of neurological functions post-SCI. GFAP was utilized to label astrocytes. The application of piezoelectric hydrogel treatment significantly attenuated glial scar formation, a major obstacle to spinal cord repair (Figures 6B, 6D).

Immature central neurons exhibit substantial axonal growth capacity, capable of sustaining robust regenerative processes. During neuronal maturation, axonal projections undergo a developmental transition from a dynamic growth-permissive state to a relatively quiescent phase characterized by stabilized "hard-wired" connectivity 49. Neuronal regeneration and axonal elongation not only establish the fundamental neuroanatomical prerequisites for circuit reconnection but also create electrophysiological substrates essential for functional recovery following SCI. Neuronal cells were identified using the Tuj-1 neuronal marker, with results demonstrating increased neuronal cell density at the lesion site following piezoelectric hydrogel intervention. Notably, neuronal regeneration and axonal elongation not only establish a fundamental neuroanatomical prerequisite for neural circuit reconnection, but also create an essential electrophysiological substrate for functional recovery post spinal cord injury (Figures 6C-D).

Endogenous NSCs (eNSCs) reside naturally within the spinal cord. Typically, following SCI, nestin-positive cells surrounding the central canal remain quiescent, whereas those outside the canal become activated. These activated cells exhibit traits of active neural stem cells (aNSCs) with robust potential for proliferation and differentiation 35. However, the disrupted microenvironment post-SCI, along with obstruction of the central canal, often results in suboptimal repair outcomes 47, 48.

To address this challenge, we employed piezoelectric stimulation to recruit eNSCs to the site of injury and provided a conducive hydrogel microenvironment that fostered their differentiation into functional neurons. One week post-SCI, we observed a substantial number of eNSCs (Nestin-positive cells) at the injury site in the pDGK-US group, while the SCI group did not exhibit this phenomenon (Figure 6E-F). This finding demonstrates that piezoelectric stimulation can effectively recruit eNSCs to migrate to the injury site. Further observations revealed that these eNSCs could successfully differentiate into functional neurons. At eight weeks post-injury, fluorescence imaging showed that appropriate piezoelectric stimulation significantly increased the number of neuronal cells at the injury site, particularly the number of Tuj-1-positive cells.

Spinal cord endothelial cells (SCECs) and spinal cord neurons are interdependent, both functionally and physically, and together they contribute to nerve regeneration and reconstruction of the microenvironment 52. We employed RECA-1 fluorescence to label SCECs and observed, under piezoelectric stimulation, increased migration of these cells to the injury site, where they formed neurovascular units in conjunction with nerve cells, thus restoring the microenvironment (Figure 6G-H).

The experimental results demonstrated that piezoelectric stimulation could promote the migration of eNSCs and activate their neurogenesis. To explore the changes in energy metabolism during this process, we detected the changes in ATP content at the injury site. The results showed that piezoelectric stimulation could increase ATP production(Figure S17). Further Western blot (WB) analysis revealed a significant upregulation of Camk2b and PGC-1α during this process (Figure 6I-K). In summary, these findings indicate that piezoelectric stimulation not only enhances energy provision but also promotes the generation of new neurons and SCECs. The convergence of these elements creates an optimized microenvironment at the injury site, thereby enhancing the efficiency and efficacy of nerve repair.

**Figure 7:**
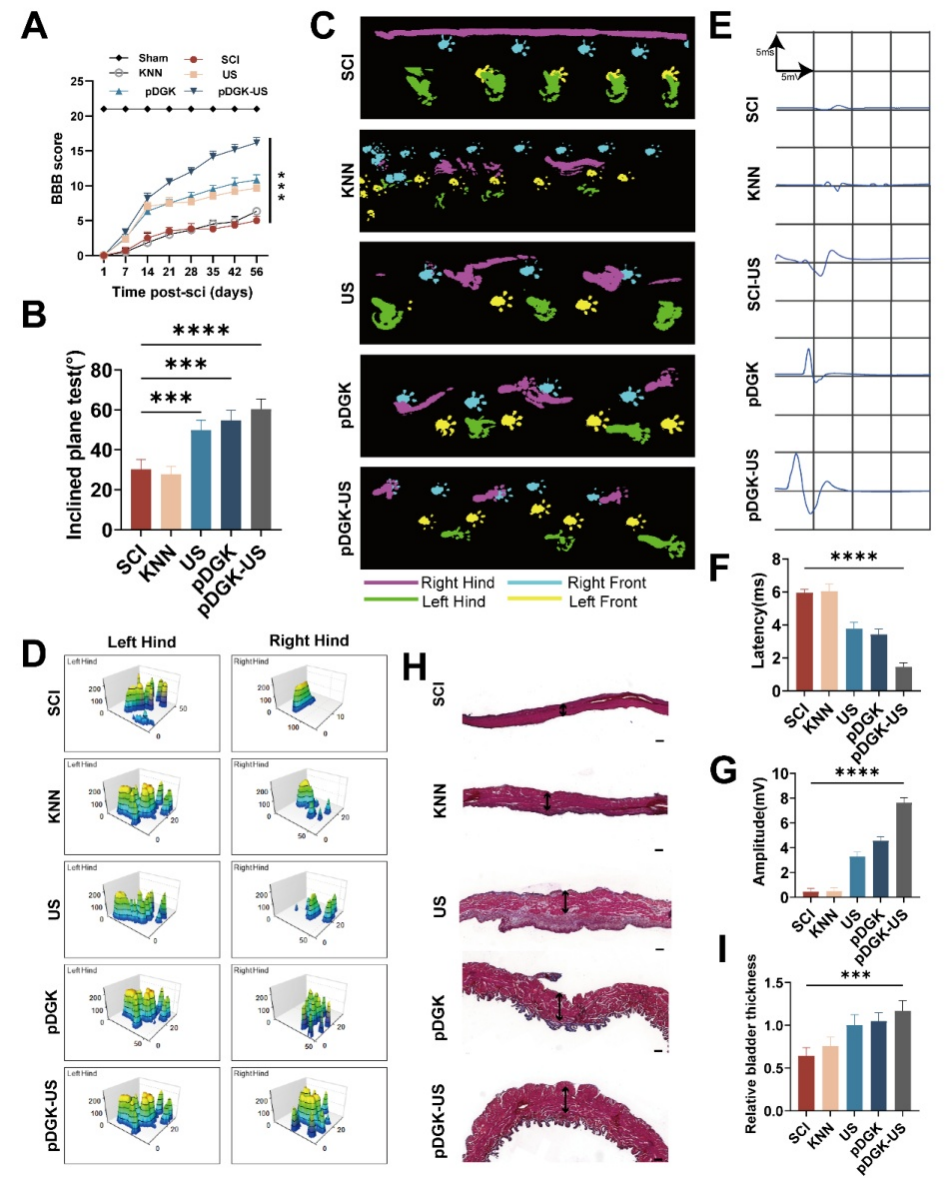
** Functional assessment of injured spinal cord repair with the piezoelectric hydrogel.** (A) Temporal analysis of BBB scores for rat hind limb function. ***p < 0.001; n = 3. (B) Results of the inclined plate test at 8 weeks, with the y-axis representing the degree of plate inclination. (C) CatWalk gait analysis at 8 weeks, highlighting the footprint of the injured side in purple. (D) Analysis of rat footprint stress, where higher peaks signify greater force, and larger areas under the curve indicate a more uniform distribution of force. (E) MEP electrophysiology in rats at 8 weeks, with each frame representing a 5 ms interval and a 5 mV amplitude. (F) Duration of MEP latency, with shorter latencies suggesting faster neural conduction and improved nerve function. ****p < 0.0001; n = 3. (G) Amplitude of the MEP, with higher amplitudes reflecting better recovery of nerve function. ****p < 0.0001; n = 3. (H) Masson's trichrome staining of rat bladder tissue, with red areas indicating smooth muscle (scale bars = 200 μm). (I) Measurement of bladder wall thickness. ***p < 0.001; n = 3.

We ensured that both histological repair and functional recovery were thoroughly assessed. Post-SCI, we performed weekly Basso, Beattie, and Bresnahan (BBB) scoring and inclined plate tests to evaluate the motor function of the rats. Compared with the other groups, the piezoelectric group exhibited significantly improved motor recovery (Figure 7A-B). Gait analysis via the CatWalk system revealed that in the SCI group, the right foot left no distinct prints and was dragged in a straight line. In contrast, the US and pDGK groups showed partial recovery of footprints, though foot dragging and poor coordination persisted. Notably, the piezoelectric group exhibited a symmetrical pressure distribution even force in the right foot, indicating superior recovery of limb function (Figure 7C-D). To confirm electrical conduction within the spinal cord, motor evoked potential (MEP) tests were performed. The piezoelectric group displayed shorter latencies and higher wave amplitudes, indicating significant improvements in both walking function and spinal cord conduction following piezoelectric treatment (Figure 7E-7G). The neurogenic bladder, a prevalent consequence of SCI, arises from disrupted electrical signaling pathways, impeding the proper initiation and completion of the voiding process. This condition can lead to urinary tract infections and severely impact patients' quality of life and health 53, 54. Therefore, restoring bladder function is crucial. Piezoelectric stimulation aids in restoring spinal cord function and effectively ameliorates bladder atrophy induced by denervation (Figure 7H-I).

## Conclusions

In this study, we engineered a biodegradable wireless piezoelectric hydrogel with the specific goal of advancing spinal cord nerve regeneration. A comprehensive set of *in vitro* and *in vivo* experiments demonstrated that piezoelectric stimulation not only augments the migration, differentiation, ATP production, and axonal growth of NSCs but also markedly improves both histological and functional recovery in SCI model rats. By creating a favorable microenvironment that stimulates neural regeneration and energy metabolism while curbing glial scar formation, our piezoelectric hydrogel promotes the formation of new neurons and vascular structures. These results highlight its potential as an innovative therapeutic strategy for SCI treatment, offering a biomimetic platform for neural tissue repair and functional restoration.

Notably, the clinical translation of this technology could synergize with emerging therapies such as human iPSC-derived cell transplantation. Recent studies have shown that hiPSCs differentiate into functional neurons within injury sites and integrate into host neural circuits, significantly improving motor recovery in preclinical models. Combining piezoelectric stimulation with hiPSCs transplantation may amplify synergistic effects by guiding stem cell differentiation and enhancing neuroplasticity. However, clinical challenges remain, including optimization of ultrasound parameters (e.g., frequency, penetration depth) for precise energy delivery, long-term biocompatibility validation in large-animal models, and standardized protocols for multimodal therapy integration. In addition, CRISPR gene-edited porcine spinal cord acellular matrix can circumvent the ethical limitations of human tissue sources. Addressing these issues through interdisciplinary collaboration will accelerate the transition from bench to bedside, ultimately bridging the gap between regenerative mechanisms and practical clinical outcomes.

## Experimental sections

### Characterization of piezoelectric hydrogels

The morphological characteristics of the piezoelectric hydrogels were examined by scanning electron microscopy (SEM), and the elemental composition was analyzed via energy-dispersive X-ray spectroscopy (EDX) using a SU8020 instrument (Japan). The crystal structure of the freeze-dried hydrogels was characterized by X-ray diffraction (XRD) on a Bruker D8 Advance system (Germany), using 2θ data ranging from 10° to 80° at room temperature with a Cu Kα radiation source.

The gelation time was assessed by placing pre-prepared DSCM pregel solutions at 37°C until no liquid movement was observed when inverted, and the time required for gelation was recorded. Subsequently, pDG and pDGK pre-gel solutions were exposed to 405 nm UV light for gelation, and the gelation time was similarly recorded. For *in vitro* degradation tests, the hydrogels were weighed to determine the initial dry weight (Wo) before being immersed in PBS solution containing lysozyme (1 mg/mL, Sigma) at 37°C. At designated time points (1, 2, 3, and 4 weeks), the samples were lyophilized and weighed (Wt) to calculate the degradation rate. Stress-strain tests were conducted using an electronic universal testing machine (Shimadzu AGS-X-50 N, Japan), with 80% strain as the endpoint. The elastic modulus was calculated by dividing the stress by the strain within the linear portion of the stress-strain curve. The piezoelectric output was measured using a piezoelectric test system comprising an electrostatic meter (Keithley 6517b, USA) and a linear motor. The linear motor applied a periodic pressure of 5 N, and the resulting voltage and current outputs under varying pressures or ultrasonic stimuli were recorded by the electrostatic meter.

### Real-time quantitative PCR (qRT-PCR)

NSC-extracted total RNA was collected from well plates using the Cell RNA Isolation Kit (RC102-01, Vazyme) and then reverse-transcribed into cDNA with the HiScript III RT SuperMix for qPCR kit (R323, Vazyme). qRT‒PCR was subsequently performed and analyzed using a QuantStudio™ 5 Real-Time PCR Detection System (Thermo Fisher, USA). The specific sequences are listed in Table S1. Target gene expression was normalized to GAPDH and analyzed via the 2^^-ΔΔCt^ method.

### Modeling and treatment of right hemisection-induced spinal cord injury

All animal experiments were performed in accordance with institutional guidelines and approved by the HuiLin ZeGu Biotechnology Co. Animal Ethics Committee (Approval No. HLZG-DELL-2023-1212-01). The animals were provided by HuiLin ZeGu Biotechnology Co., and all efforts were made to minimize suffering. The Sprague-Dawley (SD) rats (220-250 g, female) were randomly assigned to four groups of 12 rats each: SCI, US, pDGK, and pDGK-US. Anesthesia was induced with 1% sodium pentobarbital (4 mL/kg, i.p.). Laminectomy of the T9-10 vertebrae was performed to expose the spinal cord, followed by a hemisection on the right side to remove a 2-mm-long segment of the spinal cord. After achieving hemostasis, the hydrogel was implanted into the spinal cord defect, and the paravertebral muscles and skin were sutured in layers. Postoperatively, all rats were injected subcutaneously with penicillin solution for 5 consecutive days, and their bladders were massaged twice daily until reflex bladder control was restored. Additionally, the US and pDGK-US groups received ultrasound treatment for 10 minutes every morning and evening for 4 weeks.

### Immunofluorescence analysis of cells and tissues

The treated NSCs were fixed on slides, followed by membrane permeabilization, blocking with goat serum, overnight incubation with the primary antibody, room temperature incubation with the secondary antibody, DAPI nuclear staining, and mounting. Spinal cord tissues were collected and subjected to cardiac perfusion with PBS followed by 4% paraformaldehyde, and then dehydrated in a 30% sucrose solution. Frozen sections were prepared using a Leica CM1850 Cryostat (Germany). Staining procedures were performed as described above. Images were captured using a confocal laser scanning microscope (Nikon AXR NSPARC, Japan) or a 3DHISTECH Pannoramic Scan (Belgium). The primary antibodies used were GFAP (1:200, 16825-1-AP, Proteintech, China), Tuj-1 (1:200, 66375-1-Ig, Proteintech, China), CaMKII beta (1:200, 16825-1-AP, Proteintech, China), PGC1α (1:100, 66369-1-Ig, Proteintech, China), RECA-1 (1:100, sc-52665, Santa Cruz, USA). The secondary antibody was a fluorescent antibody specific to the corresponding species (Proteintech, China).

### Statistical analysis

The data are expressed as the means ± standard deviations. ANOVA with Tukey's post hoc test was used for statistical comparisons (Prism 8.0, GraphPad Software). P values less than 0.05 were considered significant (in Figure, *p < 0.05, **p < 0.01, ***p < 0.001 ****p < 0.0001).

## Supplementary Material

Supplementary methods, figures and table.
