# Supplementary material for: Biomimetic piezoelectric hydrogel system for energy metabolism reprogramming in spinal cord injury repair
Source: Theranostics. 2025 Mar 31;15(11):4955–69. doi: 10.7150/thno.108329 (PMC12036890; doi:10.7150/thno.108329)
Supplement: Supplementary file 1 — Supplementary methods, figures and table. [file thnov15p4955s1.pdf]

Supplementary files

Biomimetic piezoelectric hydrogel system for energy metabolism reprogramming in spinal cord injury repair

Guoliang Shi[1,2, #], Tianqi Su[2, #], Junyang Li[2, #], Aoao Wang[5], Gan Gao[2], Benzhang Tao[2,4], Nantian Chen[2], Lu Tian[1], Jun Yan[3,], Lingzhou Zhao[6,*], Jianning Zhang[2,*], Yantao Zhao[1,*]*

[1] Senior Department of Orthopedics, the Fourth Medical Center of PLA General Hospital; Beijing Engineering Research Center of Orthopedics Implants, Beijing, 100048, PR China

[2] Senior Department of Neurosurgery, the First Medical Center of PLA General Hospital; Medical School of Chinese PLA , Beijing, 100853, PR China

[3] Xijing 986 Hospital Department, The Fourth Military Medical University, Xi'an, 710032, PR China

[4] Tianjin Medical University, Tianjin, 300052, PR China.

[5] National Center for Orthopaedics; Beijing Research Institute of Traumatology and Orthopaedics; Beijing Jishuitan Hospital, Capital Medical University, Beijing, 100035, PR China

[6] Air Force Medical Center, The Fourth Military Medical University, Beijing, 100089, PR China

Table of contents

1 Supplementary Materials and Methods

2 Supplementary Figures

3 Supplementary References

1 Supplementary Materials and methods

1.1 Synthesis of piezoelectric hydrogel

1.1.1 DSCM pre-gel solution preparation

Spinal cords from 8-week-old Sprague-Dawley rats were collected and stored at -80°C for 48 hours. The tissues were then treated with 3% v/v Triton X-100 and 4% v/v sodium deoxycholate at 4°C, followed by repeated rinsing with phosphate-buffered

saline (PBS). Next, the tissues underwent a degreasing process using 4% v/v ethanol. Afterward, the samples were placed in a -80°C freezer and then dehydrated using a freeze dryer. The dehydrated spinal cord tissue was then pulverized. The DSCM powder was digested in an acidic solution of 0.01 M HCl containing 1 mg/mL pepsin at 25°C for 12 hours. The solution was centrifuged at 30,000 rpm for 30 minutes at 4°C to remove residual particles. The pH of the solution was adjusted to 7.4 using 1 M NaOH and isotonic equilibrium was achieved with 10× PBS to maintain a 5% w/v concentration. The pregel solution was stored at -20°C and thawed at room temperature before use [1].

1.1.2 KNN nanoparticles preparation

KNN particles were synthesized following a method described in a previous study [2]. In summary, potassium carbonate (99.0%, Aladdin, China), sodium carbonate (99.5%, Aladdin, China), and niobium oxide (99.99%, Aladdin, China) were mixed in a 1:1:2 molar ratio to prepare K0.5Na0.5NbO3 (KNN) samples. The mixture was combined with ethanol and ball milled for 12 hours, then dried. Subsequently, 8 wt% poly (vinyl alcohol) (PVA, Aladdin, China) was added to the calcined powder for granulation.

1.1.3 Piezoelectric hydrogel preparation

Porous GelMA (pGM) was provided by Engineering for Life Co. A total of 0.5 g of lyophilized pGM was dissolved in 10 mL of DSCM pre-gel solution to prepare a pDG pre-gel solution for backup. KNN nanoparticles were then added at concentrations of 0.02% w/v, 0.05% w/v, and 0.08% w/v, along with 0.25% w/v LAP. Gelation was initiated under 405 nm UV light, and the gel was further cured at 37°C. To maintain sterility, liquids were filtered through 0.22 μm Millipore filters, and solids were sterilized using cobalt-60 irradiation, all conducted in a sterile environment.

1.2 Characterization of piezoelectric hydrogel

1.2.1 DSCM characterization

Normal spinal cord tissues were collected and fixed in PFA for 48 hours, followed by dehydration in 30% sucrose solution until the tissues sank to the bottom. In contrast, DSCM was dehydrated by freeze-drying. The dehydrated tissues were embedded in

OCT gel for frozen sectioning at a thickness of 5 μm using a Leica CM1850 Cryostat (Germany), followed by HE and DAPI staining for microscopic observation with a Leica DMi8 microscope (Germany). Tissue DNA content was determined using the Qubit dsDNA Quantification Kit according to the manufacturer's instructions (Invitrogen, USA).

1.2.2 Characterization of KNN nanoparticles

KNN nanoparticles were analyzed for particle size and zeta potential using a Zetasizer Nano Zs90 (England) after ultrasonic dispersion in water. Their morphology was observed using HRTEM/SEM (FEI-Tecnai G2 F20, USA), and elemental composition was determined by EDX. The piezoelectric properties were examined using AFM (Bruker Dimension Icon, Germany), with amplitude and phase curves of KNN nanoparticles obtained in PFM mode.

1.3 In vitro studies of piezoelectric hydrogels

1.3.1 Experiments on suitable KNN nanoparticle concentration and US power

The PC-12 (CL-0481) and HUVEC (CL-0675) cells were obtained from Procell Life Science & Technology Co., Ltd. (Wuhan, China) and cultured in their respective specialized media (CM-0481, Procell; CM-0675, Procell). PC12 cells were cultured in the specialized medium with KNN nanoparticles at concentrations of 0.2% w/v, 0.5% w/v, and 0.8% w/v. The OD value at 450 nm was measured on day 3 using the Cell Counting Kit-8 (CCK8, Beyotime, China). An ultrasonic coupler was applied to the bottom of the cell culture plate, and an ultrasonic probe was used to contact the coupler and deliver ultrasonic power at 0, 0.1 W/cm^2, 0.4 W/cm^2, 0.7 W/cm^2, and 1.0 W/cm^2, respectively (Sonicator 740, METTLER TOLEDO, Switzerland). Ultrasound treatment was performed at 1 MHz, 10% duty cycle, for 5 min/day. The CCK8 assay for OD was also conducted on day 3.

1.3.2 Piezoelectric hydrogel cell activity assay

The pDGK and pDG pre-gel solutions were dispensed into 24-well plates at approximately 50 μl per well until the bottom of the wells were evenly covered. The gels were then formed under UV light for pDGK and at 37°C for pDG. PC12 cells were

seeded at a density of 10^{5} cells per well and incubated in 24-well plates. The experimental groups included sonicated pDG-US, pDGK-US, and non-sonicated pDG, pDGK. The sonicated groups underwent daily ultrasound treatment at 0.4 W/cm² for 5 minutes. After 3 days, the cells were stained using a live/dead assay kit (Beyotime, China) and observed under a fluorescence microscope for imaging.

1.3.3 Cell migration assay

After the pDG and pDGK hydrogels were placed in the lower chamber of the Transwell and cured, equal concentrations of HUVECs and PC12 cells were seeded into the upper chamber of the Transwell. Specialized media for each cell type were used in both the upper and lower chambers. After a 2-hour incubation, the sonication group underwent ultrasound treatment at 0.4 W/cm² for 5 minutes. After 24 hours, the cells were stained using a crystal violet staining kit (Beyotime, China) according to the manufacturer's instructions. Microscopic images were then captured (Olympus, Japan).

1.3.4 Neural stem cell culture and differentiation

NSC extraction was slightly modified based on a previous method described in “3D Bio-Printed Living Nerve-Like Fibers Refine the Ecological Niche for Long-Distance Spinal Cord Injury Regeneration.” Briefly, embryos were obtained from Sprague-Dawley (SD) rats at 14 days of gestation and placed in 75% ethanol. The embryos were separated, and cranial bones and blood vessels were removed in cold Hank's buffered salt solution (Solarbio, China). The hippocampus was extracted using microinstrumentation under a dissection microscope and cut into small pieces. The cells were then digested in 0.05% EDTA/trypsin (Gibco, USA) at 37°C for 10 minutes, followed by centrifugation at 200×g for 5 minutes. The cells were cultured in a 5% CO_2 incubator at 37°C using specialized media, which included 1% Glutamax (Gibco, USA), 20 ng/mL basic fibroblast growth factor (bFGF, Proteintech, China), 20 ng/mL epidermal growth factor (EGF, Proteintech, China), and Neurobasal (Gibco, USA). Half of the medium volume was replaced every 2 days. After 7 days of culture, NSC spheres were collected, stained with Nestin (1:100, sc-23927, Santa Cruz, USA) for identification, and images were captured using confocal laser scanning microscopy

(Nikon AXR NSPARC, Japan). The 200μl pDG and pDGK hydrogels were cured in 24-well plates, and NSCs were seeded at a density of $10^{\wedge 5}$ cells per well. The super-piezoelectric group was cultured for 7 days with ultrasound treatment at 0.4 W/cm² for 5 minutes per day, 1 MHz, and 10% duty cycle, while the non-piezoelectric group was cultured under normal conditions. After the hydrogel was dissolved in the lysate (EFL-GM-LS-001, Engineering for Life, China), immunofluorescence staining and RT-qPCR were performed.

1.3.5 RNA sequencing (RNA-seq) analyses

Total RNA was extracted from the treated NSCs using Trizol (Thermo Fisher, USA) for RNA-seq analysis (LC-Bio, Hangzhou, China). Bioinformatics analysis was performed using the OmicStudio online tool.

1.3.6 WB

The total protein was collected with RIPA lysis buffer containing protease and phosphatase inhibitors. The protein concentration was detected using a BCA assay kit. Next, the proteins were separated using 10% SDS-PAGE. The samples were subsequently electro-transferred onto polyvinylidene fluoride (PVDF) membranes (Millipore, MA), followed by 5% nonfat milk in PBST block for 1h at room temperature. After, the membranes were ready for incubation with primary antibodies including Tuj-1 (Proteintech, 66375-1-Ig ,1:5,000), GFAP (Proteintech, 16825-1-AP, 1:2,000), MAP2 (1:2,000), CaMKIIβ (Proteintech, 16825-1-AP, 1:1,000), PGC-1α (Proteintech, 66369-1-Ig, 1:5,000), β-actin (Proteintech, 66009-1-Ig ,1:20,000) at 4°C overnight. After rinsing three times using TBST, the samples were incubated with corresponding horseradish peroxidase-conjugated secondary antibody (1:1000, Beyotime, Shanghai, China) at room temperature for 2 h. Chemi DocXRS System (Bio-Rad, USA) was used to visualize the immunoreactive bands, and ImageJ software was used to measure the protein expression levels.

1.3.7 ATP content assay

The treated NSCs or tissues were analyzed using the Enhanced ATP Assay Kit

(Beyotime, China) following the manufacturer's instructions. Fluorescence intensity was measured using a multifunctional plate reader (Varioskan LUX, USA).

1.3.8 Calcium imaging

Groups of treated NSCs were incubated with 4 μM Fluo 4-AM (Invitrogen, USA) for 30 minutes at 37 °C according to the manufacturer's instructions. The resting fluorescence intensity (F0) was recorded using a multifunctional plate reader (Varioskan LUX, USA). Subsequently, 300 μM glutamate (Sigma, USA) was added to stimulate Ca^{2+} uptake, and the maximal fluorescence intensity (Fm) was recorded. The change in fluorescence intensity (ΔF) was calculated as Fm − F0. The ΔF/F0 ratios were calculated and normalized to represent intracellular Ca^{2+} levels.

1.4 Piezoelectric hydrogel in vivo studies

1.4.1 Functional evaluation after spinal cord injury in rats

BBB scores were assessed on postoperative day 1 and then weekly until week 8 to evaluate hindlimb function. Subsequently, the Inclined Plate Test and gait analysis were performed. The Inclined Plate Test was conducted following the procedure described in a previous study [3]. Rat tracks were recorded using an animal gait analyzer (CatWalk, Noldus). Electrophysiologic MEPs were assessed according to a previous study [4]. Amplitude and latency were measured using the PowerLab 4SP data acquisition system (Keypoint 3.02, Dantec Dynamics A/S, Copenhagen, Denmark).

1.4.2 Staining of rat bladder with Masson's trichrome staining

The bladder was fixed in 4% paraformaldehyde for 48 hours and then dehydrated in a 30% sucrose solution at 4°C. Frozen sections were subsequently prepared. Staining was performed using the Masson's Trichrome Staining Kit (Beyotime, China) according to the manufacturer’s instructions.

2 Supplementary Figures

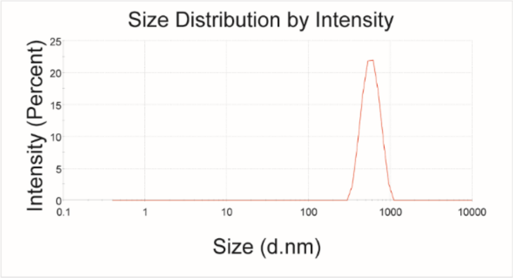

Figure S1. Average diameter of KNN nanoparticles.

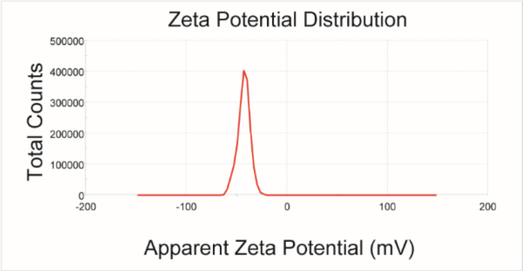

Figure S2. Zeta potential of KNN nanoparticles.

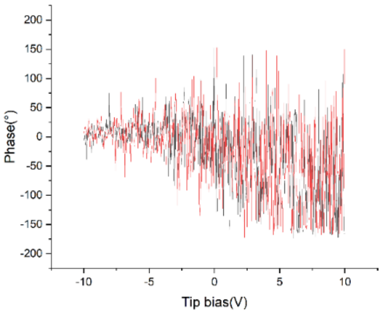

Figure S3. pDG hydrogel amplitude loop curve.

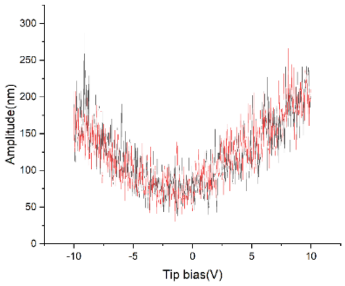

Figure S4. pDG hydrogel phase profile.

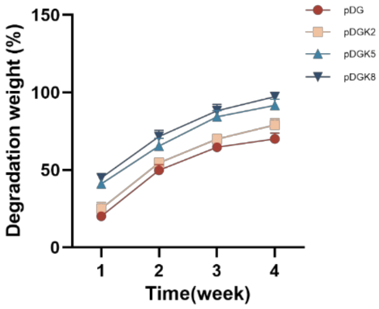

Figure S5. Degradation time of piezoelectric hydrogels.

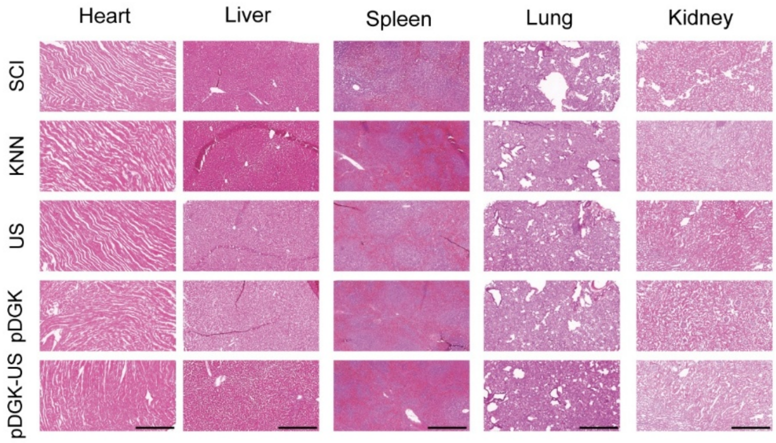

Figure S6. HE images of rat major organs tissues at 8 weeks post-sampling (scale bar = 500μm).

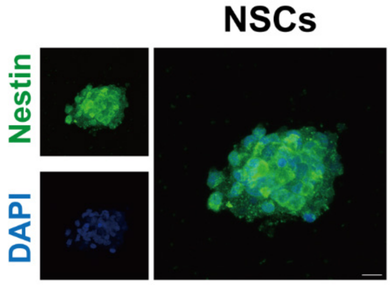

Figure S7. Diagram of hippocampal neural stem cells in fetal rats (scale bar = 50μm).

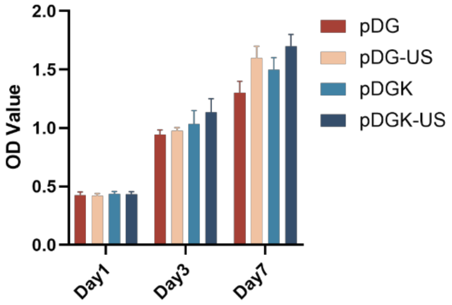

Figure S8. CCK-8 absorbance OD values for NSCs treated days with different groups. *$p < 0.05$, n = 3

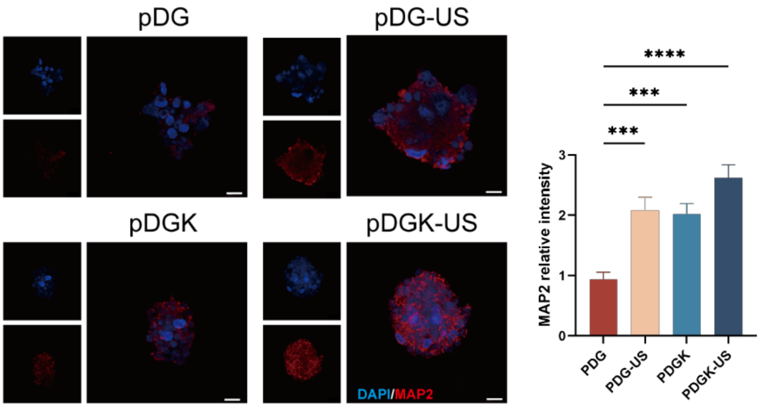

Figure S9. Neural differentiation under piezoelectric stimulation, with MAP2 labeled neurons (scale bars = 50 μm). Analysis of the fluorescence intensity of MAP2-positive cells across different groups. ***$p < 0.001$, ****$p < 0.0001$; n = 3.

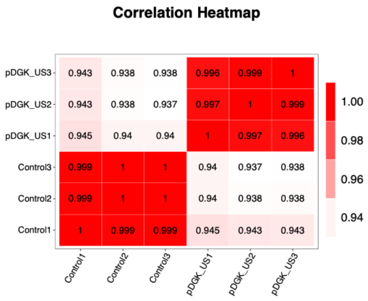

Figure S10. Inter-sample correlation coefficient analysis.

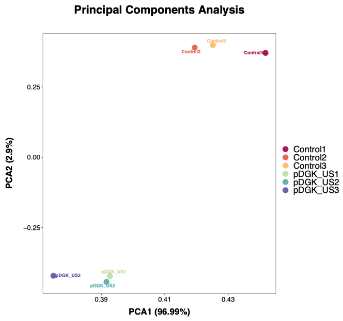

Figure S11. Principal component analysis (PCA).

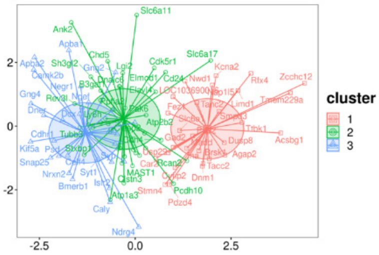

Figure S12. K-means clustering of the top 100 upregulated genes.

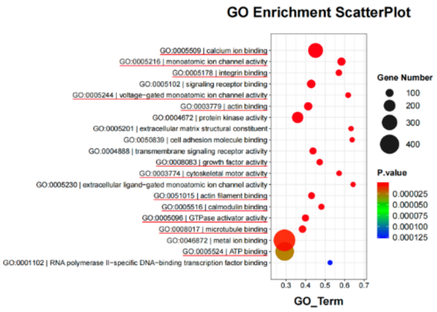

Figure S13. The bubble charts of the molecular function in the control group and pDGK-US group.

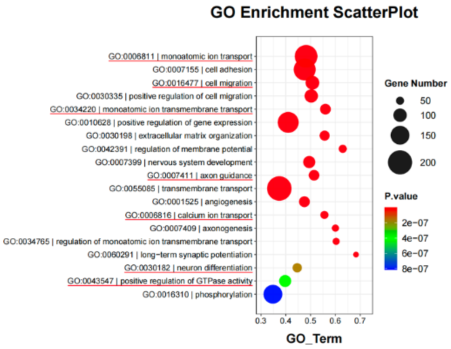

Figure S14. The bubble charts of the biological process in the control group and pDGK-US group.

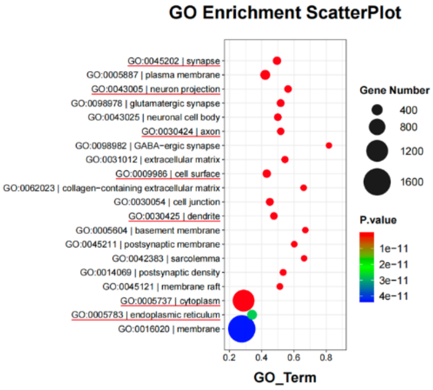

Figure S15. The bubble charts of the cellular component in the control group and pDGK-US group.

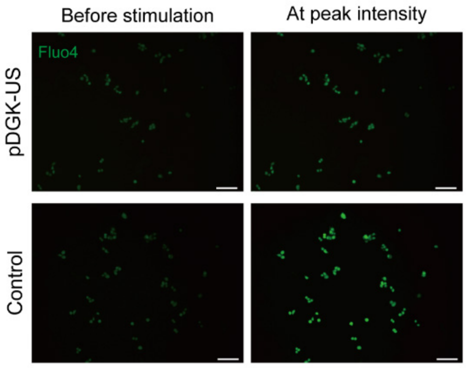

Figure S16. Pictures of representative calcium images before and after glutamate stimulation.

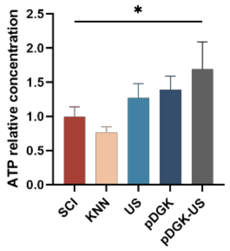

Figure S17. Relative tissue ATP content in spinal cord injury across groups. $*p < 0.05$, n = 3.

Table S1. Primer Sequences

Primer	forward	reverse
GAPDH	CAAGTTCAACGGCACAGTCAA	TGGTGAAGACGCCAGTAGACTC
Tuj-1	TGAGGCCTCCTCTCACAAGT	TGCAGGCAGTCACAATTCTC
GFAP	GCCCACCAAACTGGCTGAC	CTTGGACCGATACCACTCTTCT
MAP-2	GTTGGGCAGTGATTACTACGA	TTCAGGTAACTCGGACGGATG
Camk2b	CCCATGCCGACTGTGTTAGT	CCCTTCCCAAGACACCTGAC
PGC1α	CCTCACACCAAACCCACAGAGAAC	TTGCGACTGCGGTTGTGTATGG

3 Supplementary Reference

1. Xu Y, Zhou J, Liu C, Zhang S, Gao F, Guo W, et al. Understanding the role of tissue-specific decellularized spinal cord matrix hydrogel for neural stem/progenitor cell microenvironment reconstruction and spinal cord injury. Biomaterials. 2021; 268: 120596.
2. Yu P, Ning C, Zhang Y, Tan G, Lin Z, Liu S, et al. Bone-Inspired Spatially Specific Piezoelectricity Induces Bone Regeneration. Theranostics. 2017; 7: 3387-97.
3. Rivlin AS, Tator CH. Objective clinical assessment of motor function after experimental spinal cord injury in the rat. J Neurosurg. 1977; 47: 577-81.
4. Zhao C, Rao JS, Duan H, Hao P, Shang J, Fan Y, et al. Chronic spinal cord injury repair by NT3-chitosan only occurs after clearance of the lesion scar. Signal Transduct Target Ther. 2022;

7: 184.
